# Probing the relationship between late endogenous ERP components with fluid intelligence in healthy older adults

**DOI:** 10.1038/s41598-020-67924-4

**Published:** 2020-07-07

**Authors:** Ana C. Teixeira-Santos, Diego Pinal, Diana R. Pereira, Jorge Leite, Sandra Carvalho, Adriana Sampaio

**Affiliations:** 1 Psychological Neuroscience Laboratory – CIPsi, School of Psychology, University of Minho, Campus de Gualtar, 4710-057 Braga, Portugal; 2 Portucalense Institute for Human Development (INPP), Universidade Portucalense, Porto, Portugal

**Keywords:** Neuroscience, Physiology, Psychology

## Abstract

The world population is rapidly aging, bringing together the necessity to better understand the advancing age. This characterization may be used to aid early diagnosis and to guide individually-tailored interventions. While some event-related potential (ERP) components, such as the P300 and late positive complex (LPC), have been associated with fluid intelligence (Gf) in young population; little is known whether these associations hold for older people. Therefore, the main goal of this study was to assess whether these ERP components are associated with Gf in the elderly. Fifty-seven older adults performed a continuous performance task (CPT) and a visual oddball paradigm while EEG was recorded. Participants were divided into two groups, according to their performance in the Raven’s Advanced Progressive Matrices test: high-performance (HP) and low-performance (LP). Results showed that the HP group, compared to the LP group, had higher LPC amplitudes in the CPT and shorter P300 latencies in the oddball task, highlighting the role of ERP components as a potential electrophysiological proxy of Gf abilities in the elderly.

The world population is rapidly aging, which brings together the necessity to better understand and characterize cognitive changes due to senescence. Previous studies have shown that there are individual differences in terms of performance among the elderly, with some individuals performing at high-levels while others present very poor performances^1^. One of the cognitive abilities that is thought to decline with age, is fluid intelligence (Gf)^2,3^. Gf is the capacity of making analogies and solve original problems, independently of educational or sociocultural level^4,5^. Furthermore, Gf is a predictor of functioning in many aspects of life, such as social status, expected income, job performance, social outcomes, mortality risk, and life expectancy^6–8^. Moreover, this ability has also been associated with brain reserve, which is the individual’s brain capacity to tolerate insults and pathological processes without showing clinical deficits or symptoms^9^.

Among studies assessing the relation between brain reserve and Gf, there is strong evidence pointing out the usefulness of event-related potential (ERP) components as underlying physiological correlates of Gf^10–19^. The P300 (or P3b), a positive wave that peaks around 250–500 ms post-stimulus onset at parietal sites^20,21^, has been particularly related to Gf^14,18^. P300 is related to the “context updating”, that is, the adjustment of attentional resources called when a revision of the representation of the current environment is required^22^. More specifically, the P300 amplitude is related to the investment of attentional resources during the performance of a task, while its latency is sensitive to the time needed for stimulus detection and rating^20,23^.

Another component that has been related to Gf is the Late Positive Complex (LPC), also called Positive Slow Wave. This is a late positive wave that is largest over the centro-posterior scalp sites, occurring around 500 and 800 ms post-stimulus onset^24^. Although, there is an ongoing debate regarding the cognitive mechanisms involved in this component generation, it seems to be related to recognition memory, categorical response, memory match, decision accuracy, and maintenance of visual working memory representations^25–27^.

Finally, the P200 (or P2), a positive waveform with an anterior and central maximum distribution peaking between 100 and 250 ms after stimulus presentation^28^, has also been considered in these studies^18,29,30^. P200 is related to stimulus evaluation and context updating, and it is considered as an initial stimulus pre-classification prior to P300-related processes^28,31^.

Age-related changes in these ERP components have been reported in the literature. For example, P300 was found to be attenuated and delayed in healthy older people^32–35^ and abnormalities in this component were observed in mild cognitive impairment (MCI) and other pathological aging^36–41^. LPC absence or attenuation was also observed when comparing older with younger adults^42^ or, in contrast, an additional frontal LPC waveform in older adults that was not observed in younger ones^43^. Similarly, when comparing healthy older people with adults with MCI, a positive correlation between performance and LPC amplitude was observed in the healthy group while this relation was absent in MCI patients^38^. Similarly, P200 has also been used as a distinctive feature between younger and older adults^34,35,44^, as well as between healthy and pathological aging^38^.

While these studies have been documenting age-related changes in P300, LPC and P200 components, few studies have addressed their relationship with Gf ability. In particular, a relation between P300 and LPC amplitudes and latencies with Gf have been demonstrated in young adults^14,16,19,20,24,45–47^ and children^11,48^, however studies probing this relationship in older people are still lacking. In general, these studies have shown that Gf high-performance (HP) individuals in both children and young population present larger P300 and LPC amplitudes and shorter P300 latency when compared to low-performance (LP) individuals, except for one study performed with young women that showed an opposite result, in which HP participants exhibited a longer P300 latency than LP participants^49^. Regarding P200, whereas some studies did not observe differences in the P200 component when comparing HP and LP young adults in Gf tasks^14,48^; other studies have reported an association between the P200 latency and Gf in participants with ages between 18 and 75 years old^50^ and in young adults^30^.

Overall, there is not enough evidence about the relationship between P300, LPC and P200 and Gf in the elderly population, thus further research is needed, as it may allow the identification of neurophysiological correlates of successful aging, given that Gf is a central process in the functioning of older people^7,51,52^. Therefore, the aim of this study was to assess P200, P300 and LPC’s latencies and amplitudes during the execution of an oddball paradigm and an identical pairs-continuous performance task (CPT) as potential markers of Gf. To that end, we contrasted the P200, P300 and LPC amplitudes and latencies between HP and LP individuals. Our hypothesis was that the HP group would present higher P300 and LPC amplitudes and shorter P300 latencies when compared to the LP group, while, according to previous studies, no P200 differences were expected^14,24^. Finally, we tested the predictive relationship between these ERPs components and Gf by assessing the correlation between the ERP amplitude and latencies and the Raven’s Advanced Progressive Matrices test (RAPM) scores, as well as, by applying a regression analysis and a receiver operating characteristic (ROC) curve.

## Results

### Behavioral data

**Figure 1:**
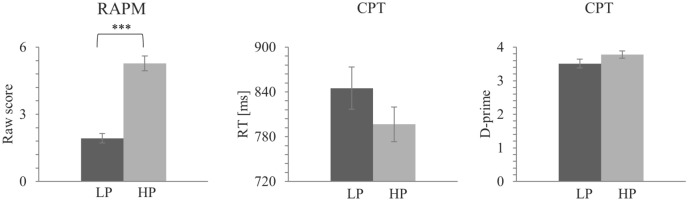
Raw mean scores in the RAPM and mean RT and D-prime for the CPT for each group*. Note.* Error bars represent standard errors. ^*p* < .1, *p < .05, **p < .01, ***p < .001. *RAPM* Raven’s Advanced Progressive Matrices, *CPT* Continuous Performance Task, *LP* low-performance, *HP* high-performance, *RT* response time.

The RAPM average score for the LP group was significantly lower than the HP group RAPM average score, *U* = 0.00, *p* < 0.01. No significant differences between the HP and LP groups were observed in CPT for response time (RT), *t*(50) = 1.29, *p* > 0.05, *d* = 0.36, 95% CI [− 25.64, 118.58] or accuracy, *t*(50) = − 1.61, *p* > 0.05, *d* = 0.03, 95% CI [− 0.60, 0.07] (see Table 1, Fig. 1 and Supplementary Table S1).

**Table 1:** Behavioral data for HP and LP in CPT task.

Behavioral performance	HP (*n* = 29)	LP (*n* = 28)
Raven	5.28 (1.79)***	1.93 (1.12)***
Correct response time (ms)	796.60 (118.40)	845.07 (142.13)
D-prime	3.78 (0.54)	3.51 (0.67)

Data are presented as mean (standard deviation).

*HP* high-performance, *LP* low-performance.

*Indicates presence of statistical difference between groups verified by independent-samples T test (***p < . 001).

### Electrophysiological data

**Figure 2:**
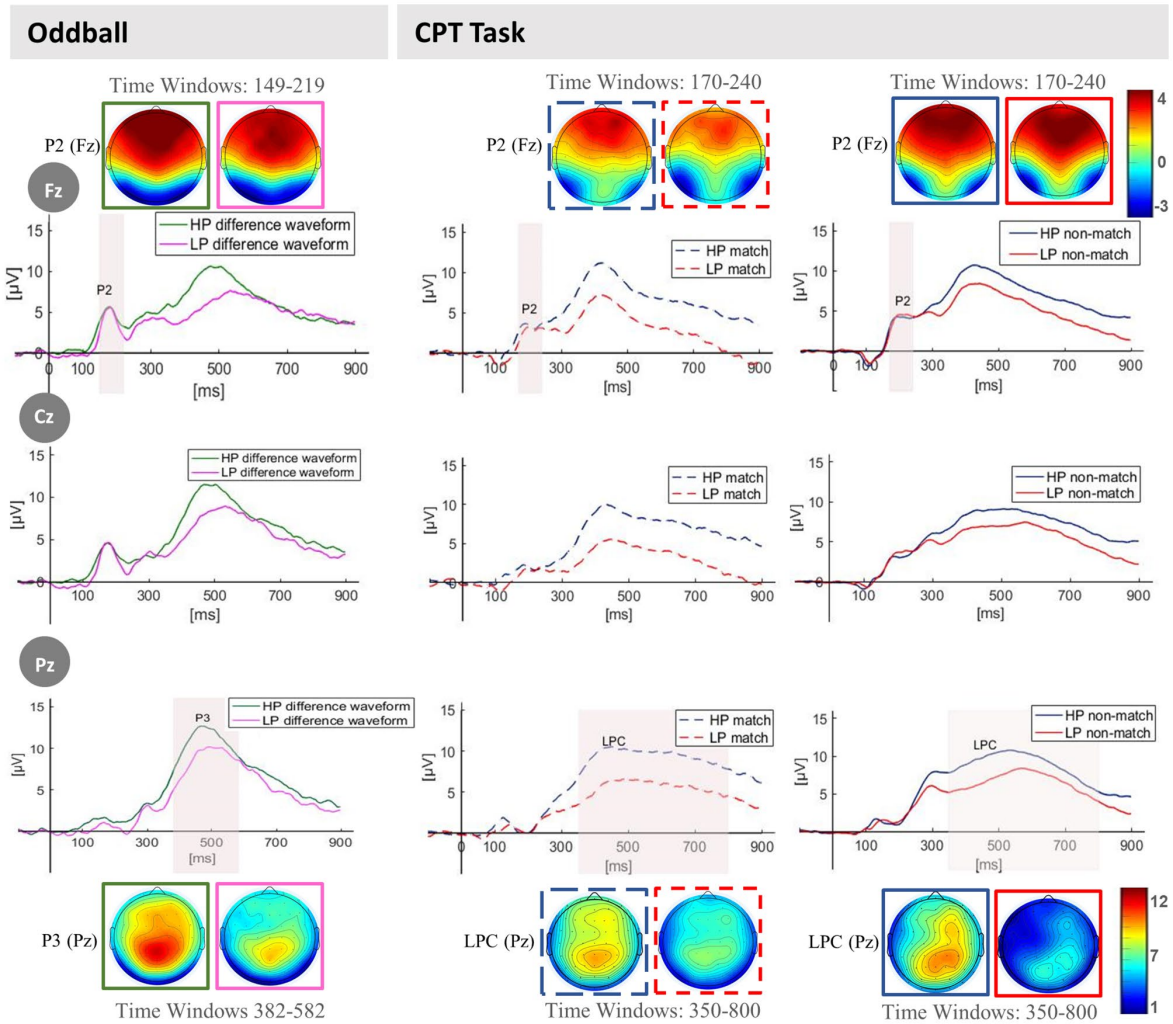
ERP waveforms (Fz, Cz and Pz electrodes) comparing LP and HP groups during CPT and oddball performance. Topographic plot of the ERP waveforms for both tasks in Fz (Top) and Pz (Bottom).

The following sections present the differences between groups in each component for the oddball task, considering the deviant minus standard difference waveform, and for the CPT, match and non-match stimuli separately (see Fig. 2 for HP and LP groups grand-average ERP waveforms, in Fz, Cz and Pz electrodes and Supplementary Table S3 for an additional ANCOVA analysis of group differences in ERP components, controlling for age).

### Group differences in the oddball task

#### P200

No significant differences (*p* > 0.05) were observed between HP and LP groups in P200 amplitude or latency in the deviant—standard difference waveforms.

#### P300

No group differences were observed for P300 amplitude in the deviant—standard difference waveforms (*p* > 0.05). P300 latency was shorter for the HP (*M* = 473.92, *SD* = 39.41) than the LP group (*M* = 503.24, *SD* = 40.71), *t*(55) = − 2.76, *p* = 0.008, *d* = − 0.73, 95% CI [− 50.58, − 8.05]; BF_10_ = 5.80.

### Group differences in match and non-match conditions of the CPT

#### P200

No significant effects were found for P200 amplitude or latency elicited by match or non-match stimuli (*p* > 0.05) in the CPT.

#### LPC

For match stimuli, LPC mean amplitude was significantly higher for the HP group (*M* = 8.56, *SD* = 4.36) in comparison with the LP group (*M* = 4.92, *SD* = 3.65) *t*(50) = 3.26, *p* < 0.001, *d* = 0.91, 95% CI [1.40, 5.88]; BF_10_ = 17.58. No significant group differences were observed for local peak latency (*p* > 0.05).

**Figure 3:**
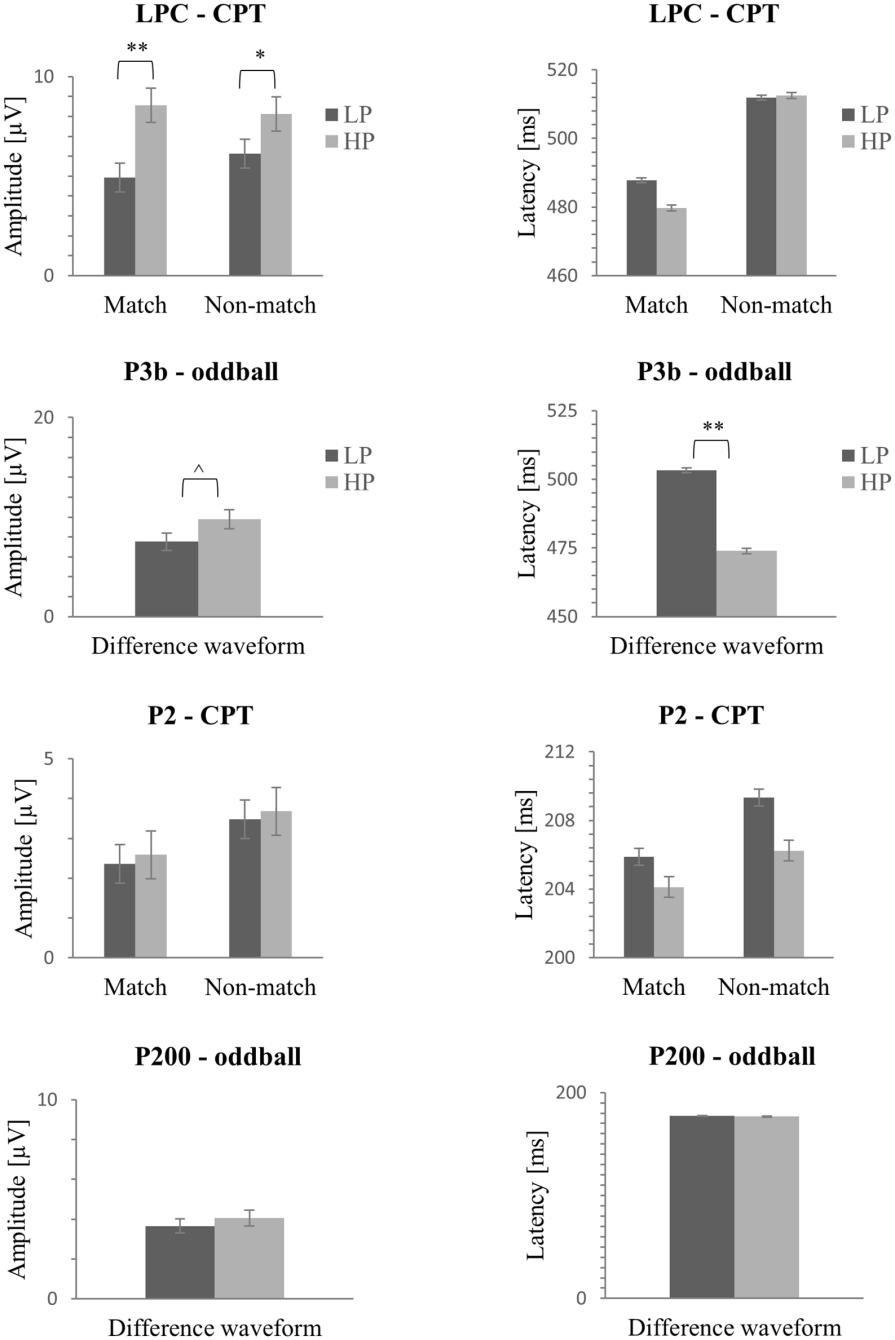
Bar graph representing LPC, P300 and P200 amplitudes and local peak latencies for match and non-match for the CPT task and deviant—standard difference waveforms for the oddball task. Error bars represent the standard error. ^*p* < .10; *p < .05; **p < .01; ***p < .001.

For non-match stimuli, the LPC amplitude was significantly larger in the HP group (*M* = 8.13, *SD* = 3.37) than in the LP group (*M* = 6.13, *SD* = 3.59), *t*(50) = 2.07, *p* = 0.04, *d* = 0.57, 95% CI [0.06, − 3.94]. However, Bayesian analysis did not support these results (BF_10_ = 1.57), there is not enough evidence available to suggest group differences in amplitude for non-match stimuli. No significant group differences were observed for LPC local peak latency (*p* > 0.05). Figure 3 shows amplitude and latency values of the aforementioned ERP components for both groups and tasks.

### Predictive analysis

Small statistically significant correlation coefficients were identified between RAPM (set II) scores and D-prime of CPT scores, P300 latency in oddball task, and LPC amplitude in match and non-match CPT conditions (see Table 2).

**Table 2:** Correlations between ERP components amplitude and latency, D-prime and RAPM scores.

Outcomes	D-prime	RAPM (set II)	P300 lat	LPC match amp	LPC non-match amp
D-prime	–	0.280*^a^	− 0.241	0.104	0.113
RAPM (set II)	0.280*^a^	–	− 0.321*^a^	0.417**^a^	0.303*^a^
P300 lat	− 0.241	− 0.321*^a^	–	− 0.39**	− 0.242
LPC match amp	0.104	0.417**^a^	− 0.39**	–	0.766**
LPC non-match amp	0.113	0.303*^a^	− 0.242	0.766**	–

*Lat* latency, *Amp* amplitude.

^a^Spearman correlations. ^*p* < .1; *p < . 05; **p < . 01; ***p < . 001.

A multiple linear regression analysis was performed to predict RAPM (set II) score based on LPC/match amplitude, LPC/non-match amplitude, and oddball’s P300 latency. Using the stepwise method, two variables were excluded from the analysis (LPC amplitude for non-match stimuli and P300 latency measured during oddball task), so only the LPC/match amplitude was entered as a predictor in the model. The model achieved statistical significance, *F*(1, 50) = 5.75, *p* = 0.02, with *R*^2 ^= 0.103. Predicted RAPM score is equal to the Eq. 2.55 + 0.17 * (LPC/match amplitude). A bootstrapping procedure with 1,000 replications (resampling with replacement), bias-corrected coefficients and confidence intervals was used to validate the model. Thus, LPC/match amplitude was observed to be a significant predictor of RAPM score.

**Figure 4:**
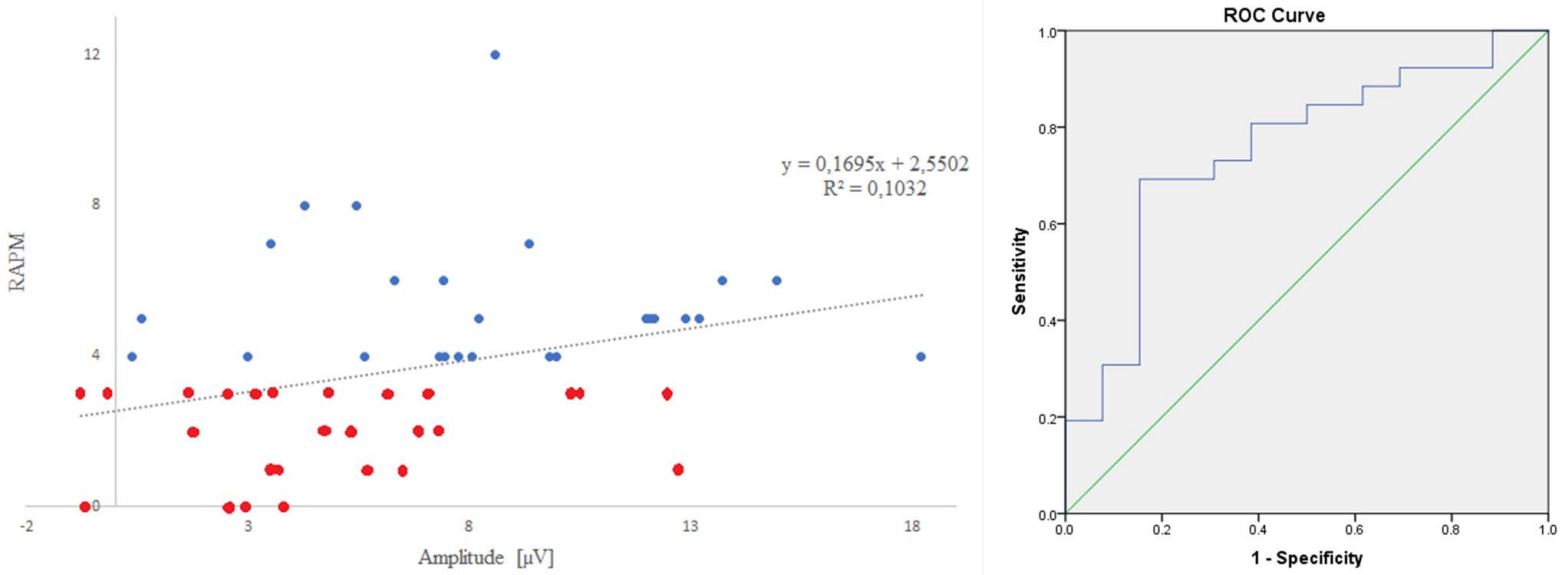
(Left) Scatter Plots showing the relationship between LPC amplitude of match stimuli and the RAPM (set 2). (Right) Receiver operating characteristic (ROC) curve for predicted scores of RAPM (set II).

The predicted RAPM score derived from the regression analysis was compared with the RAPM group state (HP vs LP) in a ROC curve (see Fig. 4). The results showed an AUC (area under the curve) of 0.75, 95% CI [0.62, 0.89]^53^, showing a moderate discriminative power of LPC/match amplitude.

## Discussion

In this work we assessed the relationship between late endogenous ERP components (i.e., P200, P300 and LPC) with Gf ability in healthy older adults. In general, between-group differences, correlations, linear regression and ROC curve analyses supported the relationship between the ERP components, recorded during CPT and oddball tasks, with Gf ability. More specifically better GF performance was associated with shorter P300 latency and higher amplitude in LPC.

Gf is a cognitive construct that has always drawn much attention, especially because of its close relationship with important life achievements, such as health in later life, mortality, daily decision-making, professional success, occupational attainment, social mobility, and school performance^54^. Besides extensive cognitive, adaptive and functional characterization of the Gf, this construct has also been studied with EEG techniques. In accordance, the literature is abundant in showing the relationship between the Gf and specific EEG signal indices. Mostly, these studies investigated the difference in late endogenous components (P200, P300 and LPC), comparing LP and HP individuals. They showed that HP individuals are faster, and present shorter ERP latencies than LP individuals. Also, HP participants have more capacity of processing information, as shown by their higher level of accuracy and larger amplitudes of late endogenous components in comparison to LP individuals^18,24,30^. However, these studies were only performed with young adults^14–19^ or children^10–13^. Our study extends this evidence to the older population.

In this study, we compared the ERP data of LP versus HP older adults and found that electrophysiological brain activity significantly differed between groups. In particular, LPC amplitudes for match-stimuli were statistically higher and oddball’s P300 latency was statistically shorter in the HP group in comparison with the LP group. Analysis of P300 amplitude in the oddball task did not achieve significance (*p* = 0.09). The difference in amplitude was more robust in LPC probably because it was elicited by the CPT task, which is more cognitively demanding than the oddball paradigm^55^. In the CPT, participants compared each stimulus with the previous one. Thus, in each stimulus, the participants must actively update the target, whereas in the oddball paradigm the participants only needed to keep track of target stimuli appearances, hence, only updating the count in 20% of the trials.

LPC has been associated with working memory maintenance processes, categorization or encoding of information^10,56–58^. Therefore, limitation in working memory processing is probably a factor underlining the observed low performance in some individuals in Gf tests, especially because working memory is a determinant factor of Gf^59^. Furthermore, and in line with our study, Gevins and Smith^24^ found significant differences in LPC amplitude elicited by a 1-back task comparing high, medium, and low performance groups, whereas no difference in latency was observed. The lack of difference in LPC latency between LP and HP groups, in this work, may be due to the inter-individual variation on the LPC waveforms^60^.

As previously mentioned, another factor that may be linked to low Gf performance is the slowing of processing speed^61,62^. Aging is associated with neural and myelination losses, as well as with a reduction in neurotransmitter levels^59^. Consequently, a decrease in processing speed accompanies the aging process, and it is supposed to be at the core of age-related cognitive decline^62,63^. P300 is related with gray matter volume in older adults and the P300 peak latency might be related to the time spent categorizing a stimulus and thus could work as an index of processing speed^57,64^. In this regard, one could infer that LP individuals present a more marked slowing of processing speed as suggested by the higher ERP latencies compared to HP participants. In fact, the LP group had a delayed peak latency in P300 in relation to the HP group.

In the current study, P200 did not differ between groups. This finding is in accordance with previous literature^14,48^. P200 is related to the evaluation of task relevant features^65^. Similar to P300, P200 amplitude increases when the target is relatively infrequent. However, unlike the P300, the P200 amplitude also varies with very simple manipulations of the perceptual features of the target stimulus (e.g., stimulus color)^23^. Superior cognitive performance is thought to be more associated with P300^20^, which might explain why the groups only differed in later components. The P200 component may be less associated with the efficiency of high-complex cognitive processes, such as those required during RAPM performance.

Our findings are also in agreement with studies with clinical populations, as they have shown late endogenous ERP components as a putative marker for general cognitive abilities^20,66^. For instance, these component latencies were found to be delayed in MCI and dementia compared to age-matched healthy peers, while the amplitude was also shown to decreased^37,67,68^. In accordance, Lai et al.^41^ suggested that P300 latency is a more sensitive tool to follow the progression of Alzheimer’s disease in comparison to neuropsychological tests.

Correlation analyses yielded a statistically significant positive correlation between LPC amplitudes and RAPM scores as well as a negative weak correlation between P300 latency and RAPM scores. These results are in line with Gevins and Smith^24^, which similarly observed a correlation between LPC amplitude elicited by a 1-back task and WAIS-R scores. In contrast with Gevins and Smith´s study, we failed to find a correlation between this ERP component and CPT accuracy, probably because CPT was an easy task for most participants and a ceiling effect was observed in participants’ behavioral performance.

Lastly, LPC amplitude of match stimuli significantly predicted RAPM scores, confirming its relationship with the Gf. The addition of the other two predictors (amplitude of non-match stimuli and P300 latency) did not improve the model. This suggests that LPC amplitude to match stimuli accounts for most of the variance, being a better predictor than the other two variables. Similarly, the Bayesian Analysis of the current study did not confirm group differences in LPC elicited by the non-match stimuli, whereas the Bayes Factor of LPC amplitude for match stimuli was much bigger than the Bayes Factor of P300 latency. Therefore, LPC amplitude for match stimuli seems to constitute a better marker compared to the other ERP components parameters. The validity of LPC amplitude to match stimuli as marker of RAPM score was also confirmed by a ROC curve, which demonstrated the predictive capacity of LPC amplitude for the discrimination between LP and HP individuals in Gf.

In this study, we have observed that LP participants displayed a decreased amplitude and an increased latency in comparison to HP individuals for LPC and P300, respectively. The same pattern was observed in studies comparing young and older adults, in which the amplitude was decreased and the latency was delayed throughout the life-span^20,69–72^. It could be postulated that more cognitively efficient elders might present a more young-like electrophysiological pattern. Therefore, future studies should address this hypothesis, contrasting HP individuals´ performance with those of younger adults. Also, they should verify the ERPs relationship with other measures of Gf. Additionally, in order to strengthen the evidence in favor of the late endogenous components as a complementary tool in the assessment and screening of elderly people^66^, future studies could assess if such ERPs work as an index of functional outcomes^73^. These studies could contribute to the development of a metric of ERPs to assess the impact of intervention protocols, such as cognitive training^74–76^.

Finally, behaviorally, accuracy in the CPT was correlated with RAPM scores, which indicates a relationship between the task used in the EEG with Gf, although no significant differences between the HP and LP groups were observed in CPT for accuracy. Additionally, shorter RT was expected in HP than in the LP group since the literature presents solid evidence of the negative relationship between processing speed and Gf^61,62^. However, group differences in RT in the CPT performance did not achieve statistical significance. It is likely that CPT is not a difficult enough task nor one demanding substantial cognitive processing. So, both groups had a high performance in the task (*d*-prime > 3), with low variability observed, which could indicate a ceiling effect for performance.

One limitation of this study was the sample size, which was unpowered to identify differences in behavioral analysis of CPT and in the P300 amplitude analysis. The dichotomization of the RAPM score in a median split could also be a limitation, since it may lead to loss of information, variability and power^77^. However, we overcame this limitation by performing a correlation and a regression analysis to corroborate our findings.

The understanding of the neurophysiological determinants of the Gf shed light on the neural mechanisms behind this cognitive dimension, which is important for the development of markers of successful aging, especially in the elderly, whose aging-related changes in brain function may arise latently in a neural process-level prior to behavioral manifestation^78^. Therefore, ERPs could be very informative of cognitive processing and could be used in complement to cognitive and neuropsychological assessment of older people, allowing early intervention when it is needed^32^. In fact, our findings highlighted the role of ERP components, in particular the LPC amplitude, as a potential electrophysiological proxy of Gf abilities in the elderly, extending prior evidence by probing such relationships that were already observed in young adults but never in healthy older adults.

## Methods

### Participants

Fifty-seven community-dwelling older adults (42 females; mean age: 68.19 ± 5.78 years old) were recruited from senior daycare centers and in sport and recreation clubs in the North of Portugal (see Supplementary Table S2 for sample characteristics). All participants were right-handed, as assessed by the Edinburgh handedness inventory^79^. They were healthy, had normal or corrected-to-normal visual (≥ 20/40 in both eyes) and auditory acuity, as well as no history of neurological or psychiatric disorders. All included participants scored above Montreal Cognitive Assessment (MoCA) cut off (of 2 standard deviation) for cognitive impairment following the normative score of the Portuguese population, according to age and educational level^80^. Participants were excluded if they scored 10 or more points in Geriatric Depression Scale^81^. The study was performed in accordance with the Declaration of Helsinki and approval was obtained from the ethics subcommittee for Life and Health Sciences of University of Minho (SECVS 012/2016). Participants gave informed consent before their inclusion in the study.

### Gf Task

The RAPM^82^ (set 1 and 2) was applied outside the EEG session. The RAPM^82^ is widely used as a standardized Gf measure due to its high loading in *g* factor, as revealed by factorial analyses studies, and high sensitivity to individual differences^4,83,84^. RAPM has been the outcome selected for assessing the effectiveness of many trials on cognitive training^85–90^. The RAPM consists of the visual presentation of 48 images, each one organized in a 3 × 3 matrix of lines and geometric shapes, wherein one of the shapes is missing. Participants were asked to select from eight options the shape that completed the matrix. A score of 1 for correct responses or 0 for errors was assigned for each item. In this experiment, only 24 items were applied (the even or odd items) with no time restriction for participants’ response.

### ERP tasks

**Figure 5:**
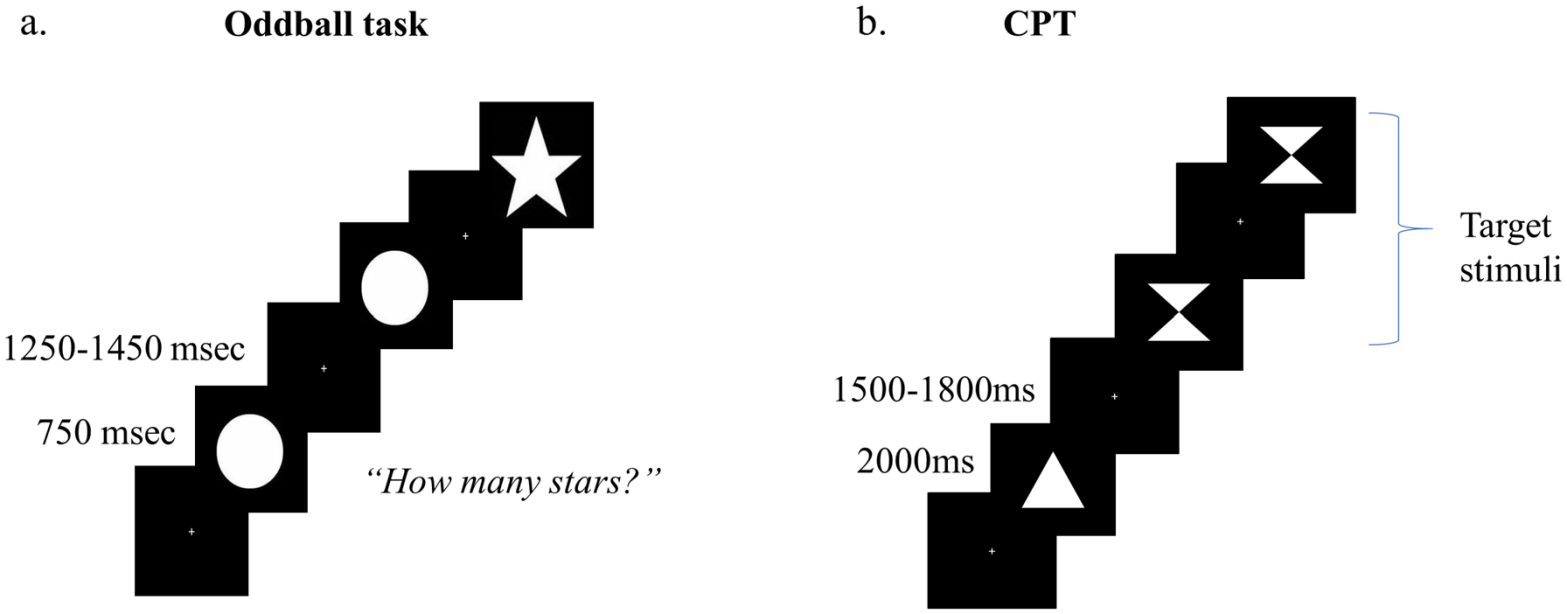
Schematic illustration of the EEG tasks. *Note.* (**a**) Oddball task. (**b**) identical pairs-continuous performance task (CPT).

The typical task used to elicit the P300 is the traditional oddball paradigm. In an active visual oddball task, two different figures are shown to the participant, one is marked as the target and is less frequently presented (deviant stimulus) than the other figure (standard stimulus), which is considered the non-target. The participants’ task is to respond (i.e., mentally counting or pressing a button) whenever they are presented with the target stimulus. In the current study, the visual oddball task (see Fig. 5a) comprised 150 trials, in which participants were randomly presented with a white circle or star on the center of a black screen (visual angle of 3.26º × 3.26º, both figures). Figures remained visible for 750 ms and were separated by a jittered interval between 1,250 and 1,450 ms. The circle was presented in 80% of the trials (standard stimulus), while the star appeared in 20% of the trials (deviant target stimulus). Participants were instructed to silently count the number of stars displayed on the screen and say the total at the end of the task. The task lasted approximately 6 min.

The CPT is another attentional task that elicits the LPC and is highly sensitive to brain dysfunction^29,91^. In this task, individuals are presented with a sequence of visual stimuli, one at a time, and they must respond when a target stimulus is presented. A version of this task is the identical pairs-CPT^91,92^, in which a target is the consecutive repetition of any item in a sequence. Identical pairs-CPT is considered to be a more complex task compared to the oddball paradigm as it depends on more controlled processing^29,93^. In the current study, during the CPT task (see Fig. 5b), participants had to decide whether the stimulus presented was the same as the one presented immediately before in a sequence (match) or not (non-match). So, they were instructed to press the key 6 (marked with a green check symbol) in a numeric keypad (CHERRY G84-4,700 Keypad) for a match stimulus, and key 4 (marked with a red ‘X’) for a non-match. The task lasted approximately 13 min, including 200 trials, in which 60 different white geometrical figures (size 4.0º × 4.0º visual angle) were presented for 2000 ms in the center of a black screen and separated by an inter-stimulus interval ranging from 1,500 to 1,800 ms. In addition, the presentation of the stimuli was pseudo-randomized, so the proportion between target and non-target trials was 1:4.

For both tasks, a fixation cross was presented in the center of the screen whenever there were no visible stimuli on screen in order to reduce ocular artifacts. Before both tasks, participants received a brief training to confirm that they understood the instructions. The order of the tasks was counterbalanced across participants.

### Procedure

The RAPM was performed the day before the EEG data collection. During EEG recording, inside an electrically shielded, soundproof room with dimmed light, participants were comfortably seated in an armchair in front of a monitor (LG ACPI × 86) placed 100 cm in front of their eyes. The Presentation software package (version 18.3; Neurobehavioral Systems, Albany, CA) was used to display stimuli and record responses.

### EEG data acquisition and analysis

Continuous EEG data band-pass filtered between 0.01 and 100 Hz were digitally recorded through a 64-channel Biosemi ActiveTwo system (Biosemi, Amsterdam, The Netherlands) at a sampling rate of 512 Hz for offline analysis. The 64 active Ag/AgCl scalp electrodes were arranged according to the international standard 10–10 system for electrode placement^94^, using a nylon head cap. Five additional active electrodes were placed in the lateral canthi of both eyes (horizontal electrooculogram—HEOG), below left eye (vertical electrooculogram—VEOG) and in right and left mastoids. As per BioSemi system design, all electrodes were referenced to the common mode sense (CMS) active electrode and grounded to a passive electrode. Further, active electrode offset was maintained below 25 mV.

EEG analysis was performed using EEGLAB (version 14.1.1)^95^ and ERPlab plugin (version v6.1.4)^96^, run in Matlab package (version 2016a). Data were passed through a digital phase-shift free Butterworth filter with the high cut-off frequency at half power (− 3 dB) set at 30 Hz (12 dB/octave roll-off) and a low cut-off frequency at half power set at 0.1 Hz (12 dB/octave roll-off). DC-bias was removed. Artifacts were rejected after visual screening for anomalies. Interpolation of visually identified noisy channels (*M* = 1.14 channels/participant; *SD* = 1.18) were done by using spherical interpolation, with a maximum of four interpolated channels. Data were referenced offline to the average of the left and right mastoids. An independent component analysis (ICA)^97^ of the data allowed the identification and deletion of components with clear ocular, muscular or noisy activity. Data were segmented in epochs from − 100 ms before stimulus presentation to 900 ms post-stimulus. Baseline was corrected with the mean activity in the 100 ms prior to stimulus onset. Artifact rejection was applied on the epoched data by using ERPlab’s functions: simple voltage threshold and sample to sample voltage threshold. Epochs were marked for rejection when the voltage was less than − 150 µV or greater than 150 µV or when the difference between consecutive samples was superior to 50 µV.

Five participants were excluded from the CPT analysis: four had more than 25% of trials rejected during artifact rejection, and one participant did not understand the task and was not able to perform it accurately. Thus, CPT analysis of P200 and LPC had 26 participants in each group. No participant was excluded from the oddball analysis. Conditions did not differ in the number of non-rejected epochs and percentage of rejected epochs (*p* > 0.05).

**Figure 6:**
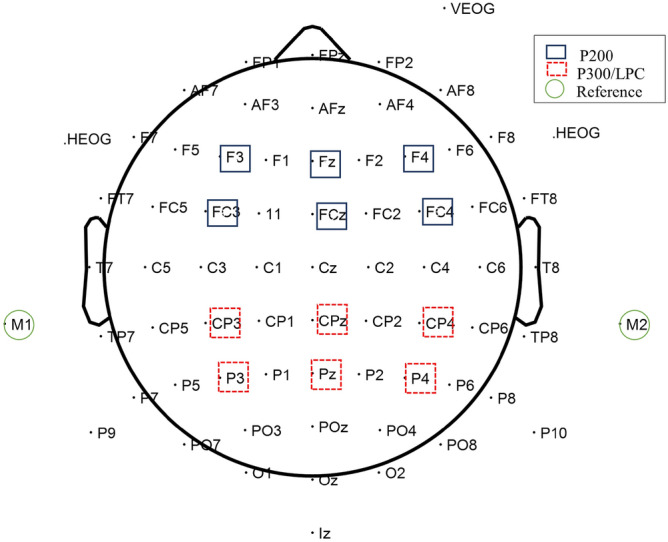
Electrode positions. *Note.* Solid blue rectangle represents electrodes used to measure the P200 component amplitude and latency. Dashed red rectangles mark electrodes used to measure the P300 and LPC components amplitude and latency. Green circles signal reference electrodes.

The following ERP waveforms were extracted for each subject: deviant—standard difference waveforms considering the oddball paradigm; and match stimuli and non-match stimuli for the CPT. For the oddball task, the P200 and P300 amplitude and latency for the difference waveforms were analyzed. For the CPT, the P200 and LPC amplitude and latency were considered separately for the conditions match and non-match. In all cases, the P300 and LPC amplitude and latency were calculated from six centro-parietal electrodes (P3, Pz, P4, CP3, CPz and CP4), while the P200 amplitude and latency were calculated from frontal and fronto-central electrodes (F3, Fz, F4, FC3, FCz, and FC4). Statistical analyses were performed on the mean values of the electrodes, at which each component was measured (see Fig. 6). Grand averages in Fz, Cz and Pz were calculated for each group for visualization purposes only.

Time windows for mean amplitude calculation were selected according to visual inspection and equally distributed around the peak latency. For the oddball task, the time windows for P300 was 382–582 ms and for P200 was 149–219 ms. For the CPT, the time windows for the LPC was 350–800 ms and 170–240 ms for the P200. In this task, only epochs corresponding to correct responses occurring between 200 and 3,500 ms after the onset of a matching stimulus entered the analysis.

### Statistical data analyses

Statistical analyses were performed on the Statistical Package for the Social Sciences (SPSS) Version 24.0 (SPSS Inc., Chicago, IL, USA), adopting an alpha level of 0.05. Only significant results were reported (for overall results, see Supplementary, Table S4). Effect sizes were calculated through Cohen’s d (*d*). Participants were divided in HP, if they performed equal or above the median of raw scores in RAPM (set II) (*Md* = 4), and LP, if their performance was below the median.

First, we verified group differences in the raw scores of the RAPM. Then, the behavioral analysis of EEG tasks was performed only for the CPT task, as in the oddball task the participants’ output was restricted to the total number of stars counted during the task. The outcomes considered were reaction time (RT) from stimulus onset to button press (considered only for correct responses) and accuracy (D-prime)^98,99^. Two-tailed student’s *t*-tests for unpaired groups were performed comparing HP with LP group’s behavioral outcomes as well as the mean amplitude and local peak latency for each ERP component. When normality was not verified, the Mann–Whitney U‐test was used. Results were considered significant at *p* < 0.05. We also confirmed between-group results with Bayesian analysis (see Supplementary Table S5) run in JASP software, version 0.9.2^100^. Bayesian results were considered substantial when BF were bigger than 3 and the 95% credible interval did not include zero.

Additionally, bivariate correlation analyses were performed to test the association between the ERP components that were significant in the LP vs HP analysis (i.e., LPC/match mean amplitude; LPC/non-match mean amplitude; P300 peak latency) and RAPM scores (Table 2). When both variables in the analysis were normally distributed, we used Pearson’s correlation coefficient, otherwise Spearman’s correlation coefficient was performed. An additional multiple linear regression analysis was conducted to assess if those ERP components amplitude and latency could predict Gf. Assumptions for linear regression were checked and the stepwise method was performed with the ERP components’ parameters as predictors, and RAPM scores in set 2 as dependent variables. There was one outlier in the total sample regarding RAPM scores, however a sensitive analysis indicated no change in the results when this participant was excluded. Therefore, we considered data derived from this participant in the analysis. Finally, we used the ROC curves to assess the predictive discrimination of ERP components to identify HP and LP individuals in Gf^101^.

## Data availability

Datasets are available under reasonable request.

## Supplementary information

Supplementary information 1
