# Author Correction: Probing the relationship between late endogenous ERP components with fluid intelligence in healthy older adults

**DOI:** 10.1038/s41598-021-86895-8

**Published:** 2021-03-26

**Authors:** Ana C. Teixeira-Santos, Diego Pinal, Diana R. Pereira, Jorge Leite, Sandra Carvalho, Adriana Sampaio

**Affiliations:** 1 Psychological Neuroscience Laboratory – CIPsi, School of Psychology, University of Minho, Campus de Gualtar, 4710‑057 Braga, Portugal; 2 Portucalense Institute for Human Development (INPP), Universidade Portucalense, Porto, Portugal

Correction to: *Scientific Reports* 10.1038/s41598-020-67924-4, published online 07 July 2020

The Acknowledgements section in this Article is incomplete.

“We thank Câmara Municipal de Vila Nova de Famalicão (Dr. Rui Baptista and Bruno Gomes), Associação Gerações (Dr. Cristiana Oliveira, Clara and Daniela Silva), Santa Casa da Misericórdia de Barcelos (Dr. Ricardo Vieira and Dr. Helder Longras), and Fundação Bomfim (Dr. Raquel Polonia) for hosting the study; all the volunteers for their participation, as well as, Silvia Alves, Carla Barros, Anabela Fernandes and our colleagues from the Psychological Neuroscience Laboratory for all the help during data collection and recruitment. This work was supported by the Portuguese Foundation for Science and Technology (FCT) [Doctoral Grants No. SFRH/BD/80965/2011 (awarded to ACT) and No. PD/BD/105964/2014 (awarded to DRP)] and by the Bial Foundation (Grant Number #286/16). It was conducted at the Psychology Research Centre (PSI/01662), School of Psychology, University of Minho, and supported by the Portuguese Foundation for Science and Technology and the Portuguese Ministry of Science, Technology and Higher Education (Grant Number UID/PSI/01662/2019), through the national funds (PIDDAC). DP was supported by FCT (Grant Number SFRH/BPD/120111/2016). SC was funded by the FCT (Grant Number IF/00091/2015) and COMPETE 2020 (Grant Number PTDC/PSI-ESP/29701/2017).”

should read:

“We thank Câmara Municipal de Vila Nova de Famalicão (Dr. Rui Baptista and Bruno Gomes), Associação Gerações (Dr. Cristiana Oliveira, Clara and Daniela Silva), Santa Casa da Misericórdia de Barcelos (Dr. Ricardo Vieira and Dr. Helder Longras), and Fundação Bomfm (Dr. Raquel Polonia) for hosting the study; all the volunteers for their participation, as well as, Silvia Alves, Carla Barros, Anabela Fernandes and our colleagues from the Psychological Neuroscience Laboratory for all the help during data collection and recruitment. This work was supported by the Portuguese Foundation for Science and Technology (FCT) [Doctoral Grants No. SFRH/BD/80965/2011 (awarded to ACT) and No. PD/BD/105964/2014 (awarded to DRP)] and by the Bial Foundation (Grant Number #286/16). It was conducted at the Psychology Research Centre (PSI/01662), School of Psychology, University of Minho, and supported by the Portuguese Foundation for Science and Technology and the Portuguese Ministry of Science, Technology and Higher Education (Grant Number UID/PSI/01662/2019), through the national funds (PIDDAC). AS and DP were supported by FCT with the grants NORTE-01-0145-FEDER-032152 (PTDC/PSI-GER/32152/2017) and POCI-01-0145-FEDER-028682 (PTDC/PSI-GER/28682/2017). DP was supported by FCT (Grant Number SFRH/BPD/120111/2016). SC was funded by the FCT (Grant Number IF/00091/2015) and COMPETE 2020 (Grant Number PTDC/PSI-ESP/29701/2017).”

