# Supplementary material for: Probing the relationship between late endogenous ERP components with fluid intelligence in healthy older adults
Source: Sci Rep. 2020 Jul 7;10:11167. doi: 10.1038/s41598-020-67924-4 (PMC7341872; doi:10.1038/s41598-020-67924-4)
Supplement: Supplementary file 1 — Supplementary information 1 [file 41598_2020_67924_MOESM1_ESM.docx]

Probing the relationship between late endogenous ERP components with fluid intelligence in healthy older adults

Ana C. Teixeira-Santos, Diego Pinal, Diana R. Pereira, Jorge Leite, Sandra Carvalho, Adriana Sampaio*

**Appendix A. Supplementary material**

*Table S1.*

*Results of Bayesian Independent Samples t-tests Analysis of Group Differences (LP versus HP) in Response time (RT) and D-prime for CPT.*

|  |  | | | | | |  |
| --- | --- | --- | --- | --- | --- | --- | --- |
|  | | BF₁₀ | | BF_01_ | error % | | 95% CI |
| Response time |  | 0.575 |  | 1.739 | 0.021 |  | [- 0.19, 0.83] |
| D-Prime |  | 0.797 |  | 1.254 | 0.006 |  | [- 0.92, 0.13] |

*Note.* BF = Bayes Factors; CPT = Continuous Performance Task. Bayes factors were determined by Bayesian two-tailed independent t-tests.

*Table S2*.

Sample Demographic Characteristics

| Demographic characteristic | LP group (*n* = 28) | |  | HP group (*n* = 29) | |
| --- | --- | --- | --- | --- | --- |
|  | *M* | *SD* |  | *M* | *SD* |
| Age (years) | 69.64* | 5.53 |  | 66.79* | 5.77 |
| Education (years) | 6.29 | 3.93 |  | 8.90 | 5.91 |
| MoCA | 23.97** | 2.72 |  | 21.50** | 2.43 |
| Gender (Male/Female) | 7/21 |  |  | 8/21 |  |

Note. *Indicates presence of statistical difference between groups verified by Mann-Whitney test (age and years of formal education), independent-samples t-test (MoCA), and chi-square (gender) (*p <. 05, **p <. 01). HP = High-performance; LP = Low-performance.

As frequentist analysis suggested group differences in age (see Table S2), we run analyses of covariance (ANCOVA) controlling for age (see table S3), but the results remained the same as the Student’s *t*-test analyses reported in the manuscript. Likewise, Bayesian analysis yielded no group differences in both variables: age and education (BF_age_ = 1.188; BF_education_ = 1.29).

Table S3.

*ANCOVA results of Group Difference (LP versus HP) in ERP components Amplitude (amp) and Latency (lat), Controlling for Age.*

|  | Component | Condition | Amp/ lat | Age effect | Corrected model |
| --- | --- | --- | --- | --- | --- |
|  |  |  |  |  |  |
| CPT | LPC | Match | Amplitude | *F*(1,49) = 0.87, *p* = 0.36 | ***F*(2,49)= 5.74, *p* = 0.01** |
|  | LPC | Match | Latency | *F(*1,49) = 0.50, *p* = 0.48 | *F*(2,49) = 0.36, *p* = 0.70 |
|  | LPC | Non-match | Amplitude | ***F*(1,49) = 5.54, *p* = 0.02** | ***F*(2,49) = 5.10, *p* = 0.01** |
|  | LPC | Non-match | Latency | *F*(1,49) = 0.64, *p* = 0.43 | *F*(2,49) = 0.32, *p* = 0.73 |
|  | P2 | Match | Amplitude | *F*(1,49) = 0.06, *p* = 0.80 | *F*(2,49) = 0.327, *p* = 0.72 |
|  | P2 | Match | Latency | *F*(1,49) = 0.08, *p* = 0.78 | *F*(2,49) = 0.82, *p* = 0.45 |
|  | P2 | Non-match | Amplitude | *F*(1,49) = 0.81, *p* = 0.37 | *F*(2,49) = 0.73, *p* = 0.49 |
|  | P2 | Non-match | Latency | *F*(1,49) = 0.39, *p* = 0.53 | *F*(2,49) = 0.20, *p* = 0.82 |
|  |  |  |  |  |  |
| Oddball | P3 | Wave difference | Amplitude | *F*(1,54) = 0.66, *p* = 0.42 | *F*(2,54) = 1.81, *p =* 0.173 |
|  | P3 | Wave difference | Latency | *F*(1,54) = 0.43, *p* = 0.52 | ***F*(2,54) *=* 3.99, *p* = 0.02** |
|  | P2 | Wave difference | Amplitude | *F*(1,54) = 0.23, *p* = 0.33 | *F*(2,54) = 0.96, *p* = 0.33 |
|  | P2 | Wave difference | Latency | *F*(1,54) = 3.88, p= 0.05 | *F*(2,54) = 1.95, *p* = 0.15 |

*Note.* CPT = Continuous Performance Task. Amplitude in μV, Latency (Lat) in milliseconds. Bold *p*-values indicate significant values (*p* >.05).

Table S4.

*Results of t-test Analysis of Group Difference (LP versus HP) in ERPs Components Amplitude (amp) and Latency (lat)*

|  | Component | Condition | Amp/Lat | Mean (*SD*) LP | Mean (*SD*) HP | *t* test results | *p*-value | Cohen’s *d* | 95% CI |
| --- | --- | --- | --- | --- | --- | --- | --- | --- | --- |
| CPT | LPC | Match | Amplitude | 4.92 (3.65) | 8.56 (4.36) | *t*(50)=3.26 | **<0.01** | 0.91 | [1.40, 5.88] |
|  | LPC | Match | Latency | 487.84 (64.66) | 479.67 (59.95) | *t*(50)=-0.47 | 0.64 | -0.13 | [-42.91, 26.56] |
|  | LPC | Non-match | Amplitude | 6.13 (3.59) | 8.13 (3.37) | *t*(50)=2.07 | **0.04** | 0.57 | [0.06, 3.94] |
|  | LPC | Non-match | Latency | 511.92 (72.27) | 512.53 (59.48) | *t*(50)=0.03 | 0.973 | <0.01 | [-36.29, 37.51] |
|  | P2 | Match | Amplitude | 2.33 (2.57) | 2.95 (3.12) | *t*(50)=0.78 | 0.441 | 0.22 | [-0.98, 2.21,] |
|  | P2 | Match | Latency | 205.01 (17.09) | 204.75 (17.80) | *t*(50)=-0.06 | 0.956 | -0.02 | [-9.99, 9.45] |
|  | P2 | Non-match | Amplitude | 3.45 (2.85) | 4.03 (2.81) | *t*(50)=0.74 | 0.46 | 0.21 | [-1.00, 2.16] |
|  | P2 | Non-match | Latency | 209.38 (16.95) | 206.43 (18.11) | *t*(50)=-0.61 | 0.55 | -0.17 | [-12.72, 6.82] |
|  |  |  |  |  |  |  |  |  |  |
| Oddball | P3 | Wave difference | Amplitude | 7.54 (4.60) | 9.78 (5.17) | *t*(55)=1.73 | 0.09 | 0.46 | [-0.36, 4.84] |
|  | P3 | Wave difference | Latency | 503.24 (40.71) | 473.92 (39.41) | *t*(55)=-2.76 | **0.008** | -0.73 | [-50.58, -8.05] |
|  | P2 | Wave difference | Amplitude | 3.66 (1.83) | 4.05 (2.14) | *t*(55)= -0.16 | 0.87 | 0.20 | [-0.66, 1.45] |
|  | P2 | Wave difference | Latency | 177.32 (16.01) | 176.70 (12.98) | *t*(55)=-0.75 | 0.46 | 0.04 | [-8.34, 7.11] |

*Note.* CPT = Continuous Performance Task. Amplitude in μV, Latency (Lat) in milliseconds. LP = Low-performance; HP = High-performance. Bold *p*-values indicate significant values (*p* > .05).

**Bayesian T-Tests**

Table S5.

*Results of Bayesian Independent Samples t-tests Analysis of Group Differences (LP versus HP) in ERP Components Amplitude (amp) and Latency (lat)*

|  | **Component** | | **Condition** | **Amp/Lat** | **BF_10_** | **BF_01_** | **Error%** | **95% CI** |
| --- | --- | --- | --- | --- | --- | --- | --- | --- |
| CPT | LPC | Match | | Amplitude | **17.58** | 0.057 | 7.159e-5 | **[-1.376, -.242]** |
|  | LPC | Match | | Latency | 0.305 | 3.277 | 0.016 | [-.378, .618] |
|  | LPC | Non-match | | Amplitude | 1.568 | 0.638 | 0.001 | [-1.031, 0.031] |
|  | LPC | Non-match | | Latency | 0.278 | 3.592 | 0.016 | [-.501, .481] |
|  | P2 | Match | | Amplitude | 0.357 | 2.802 | 0.017 | [-.668, .313] |
|  | P2 | Match | | Latency | 0.279 | 3.589 | 0.016 | [-.481, .519] |
|  | P2 | Non-match | | Amplitude | 0.349 | 2.867 | 0.017 | [-.675, .330] |
|  | P2 | Non-match | | Latency | 0.324 | 3.088 | 0.016 | [-.359, .635] |
|  |  |  | |  |  |  |  |  |
| Oddball | P3 | Wave difference | | Amplitude | 0.917 | 1.091 | 0.013 | [-.892, .099] |
|  | P3 | Wave difference | | Latency | **5.795** | 0.173 | 3.968e-6 | **[.131, 1.189]** |
|  | P2 | Wave difference | | Amplitude | 0.338 | 2.957 | 0.007 | [-.644, .303] |
|  | P2 | Wave difference | | Latency | 0.271 | 3.691 | 0.007 | [-.425, .515] |

*Note.* CPT = Continuous Performance Task. BF = Bayes factors. CI = Credible interval. Bayes factors were determined by Bayesian two-tailed independent *t*-tests. Substantial results are marked in bold.
